# Guidelines for treating child and adolescent obesity: A systematic review

**DOI:** 10.3389/fnut.2022.902865

**Published:** 2022-10-12

**Authors:** Louise Tully, Niamh Arthurs, Cathy Wyse, Sarah Browne, Lucinda Case, Lois McCrea, Jean M. O’Connell, Clodagh S. O’Gorman, Susan M. Smith, Aisling Walsh, Fiona Ward, Grace O’Malley

**Affiliations:** ^1^Obesity Research and Care Group, School of Physiotherapy, RCSI University of Medicine and Health Sciences, Dublin, Ireland; ^2^W82GO Child and Adolescent Obesity Service, Children’s Health Ireland at Temple Street, Dublin, Ireland; ^3^School of Public Health, Physiotherapy and Sports Science, University College Dublin, Dublin, Ireland; ^4^St. Columcille’s Hospital Weight Management Service, St.Vincent’s University Hospital, Dublin, Ireland; ^5^School of Medicine, University of Limerick, Limerick, Ireland; ^6^Department of Paediatrics, University Hospital Limerick, Limerick, Ireland; ^7^Discipline of Public Health and Primary Care, Trinity College Dublin, Dublin, Ireland; ^8^Department of Epidemiology, Division of Population Health Sciences, RCSI University of Medicine and Health Sciences, Dublin, Ireland; ^9^Department of Clinical Nutrition and Dietetics, Children’s Health Ireland at Crumlin, Dublin, Ireland

**Keywords:** clinical practice guidelines, paediatric obesity, weight management, childhood obesity treatment, obesity management, bariatric, physical function, medical nutrition therapy

## Abstract

Obesity is a chronic disease that compromises the physical and mental health of an increasing proportion of children globally. In high-income countries, prevalence of paediatric obesity is increasing faster in those from marginalised populations such as low-income households, suggesting the disease as one that is largely systemic. Appropriate treatment should be prioritised in these settings to prevent the development of complications and co-morbidities and manage those that already exist. An array of clinical practice guidelines are available for managing overweight and obesity in children and adolescents, but no systematic review has yet compared their quality or synthesised their recommendations. We aimed to narratively review clinical practice guidelines published in English for treating child and adolescent obesity, to identify the highest quality guidelines, and assess similarities, conflicts, and gaps in recommendations. We systematically searched academic databases and grey literature for guidelines published. We used the AGREE II tool to assess the quality, and identified nine high quality guidelines for inclusion in a narrative review of recommendations. Guidelines predominantly recommended the delivery of multi-component behaviour-change interventions aimed at improving nutrition and physical activity. Treatment outcomes were generally focussed on weight, with less emphasis on managing complications or improving quality-of-life. There was no evidence-based consensus on the best mode of delivery, setting, or treatment format. The guidelines rarely included recommendations for addressing the practical or social barriers to behaviour change, such as cooking skills or supervised physical activity. There is insufficient evidence to evaluate pharmaceutical and surgical interventions in children, and these were generally not recommended. It should be noted that this review addressed documents published in English only, and therefore the included guidelines were applicable predominantly to high-resource settings.

## Introduction

Obesity in children and adolescents has become an international public health issue, with most countries reporting an increased prevalence over the last four decades. According to global estimates, there were 250 million children and adolescents overweight, and a further 124 million with obesity in 2016 (1). Furthermore 1 in 4 (25%) children with obesity aged 6–9 years across 21 European World Health Organisation (WHO) member states have severe obesity (2). In many high-income countries where the increase in prevalence of obesity has stabilised somewhat, this pattern has not been seen for children experiencing low socioeconomic status, whereby the trend has continued upward (3–5). Further, emerging evidence suggests that this plateau may be reversing globally as a result of the COVID-19 pandemic and associated lockdown restrictions (6). There is widespread consensus among clinicians and scientists that childhood obesity should be recognised and treated as a chronic disease (7), and that it is multifactorial, driven largely by the environment and sociocultural systems, in combination with genetic and biological factors (8).

In 2012, the World Health Assembly endorsed an implementation to specifically target childhood obesity, aiming to halt further increases by 2025 (Resolution 65.6) (9). Countries across the world are implementing public health and clinical guidelines for prevention and treatment of obesity in their children but it is already considered unlikely that any of the 191 countries that participated will meet their 2025 target (1).

When the onset of obesity occurs during childhood or adolescence it can cause health sequelae in both the short- and longer term (10). The physical and psychosocial complications of obesity can extend beyond increased body weight or a large body habitus. Obesity in childhood is associated with metabolic, cardiorespiratory (11, 12), cardiovascular (13), hepatic (14), gastrointestinal (15), genitourinary (16), and musculoskeletal complications (17), as well as mental health conditions (18, 19). It can adversely affect the child’s capacity to play or to engage in physical activity and in turn can limit participation (20, 21), and expose vulnerable children to relentless social stigma and bullying (19, 22). Children with obesity have been reported to have lower academic performance (23, 24), cognitive capacity (25, 26) and self-esteem (27, 28), compared to their healthy-weight counterparts, although many complex interacting factors might confound these relationships, such as sleep quality, weight bias from teachers, or nutritional status (23). In many cases, obesity and related complications will progress into adulthood (29), increasing the risk of additional chronic diseases including cardiovascular disease (30–32), stroke (33, 34), osteoarthritis (35–37), and certain cancers (38, 39).

The increasing prevalence and long-term health consequences of childhood obesity have led many governments and regulatory bodies to develop and implement preventative interventions at the population level in order to arrest the early development of obesity during childhood. In tandem, there is increasing focus on the need to offer treatment to children and adolescents diagnosed with clinical obesity where the excessive or ectopic accumulation of adipose tissue impairs health in early life. During childhood and adolescence, holistic obesity management programmes are widely considered the cornerstone of treatment for overweight and obesity, as systematic reviews have demonstrated behaviour change techniques alongside medical nutrition therapy and exercise support to be effective (40, 41). For those with more severe obesity, in addition to conservative treatment, pharmacotherapy and surgical approaches may be needed, though there is a dearth of evidence for their long term efficacy and safety (42, 43).

Clinical practice guidelines are evidence-based recommendations used by health professionals to inform decision-making and to improve the quality or type of care offered to patients. The U.S. Institute of Medicine (now the National Academy of Medicine) defined clinical practice guidelines as “statements that include recommendations intended to optimise patient care that are informed by a systematic review of evidence and an assessment of the benefits and harms of alternative care options” (44). Guidelines can facilitate patient education and empowerment, can assist configuration of health services or can be used to compare different treatment approaches. Whilst a variety of guidelines exist for treatment of child and adolescent obesity by setting and discipline, no systematic review has yet compared their quality or synthesised their recommendations.

Given the diverse and manifold complications of childhood obesity on the health and social functioning of children and adolescents, in addition to the relatively recent emergence of this public health challenge, usable evidence-based clinical guidelines are imperative in order to implement high quality, effective and equitable obesity interventions in health and social care services. Implementation of evidence-based interventions should support management of obesity at an early stage, benefitting the individual child with obesity, and reducing the social and economic costs of managing their obesity in adulthood. These should include clinical assessments that consider the systemic nature of obesity in order to inform appropriate interventions which are tailored to the child and family, allowing a holistic approach.

The aim of this paper is to assess the quality of published international clinical practice guidelines for treating child and adolescent obesity, and narratively synthesise the high quality, contemporary recommendations to aid decision making related to paediatric bariatric practice. Further, we aim to highlight best practice and to recommend how guidelines might be used to guide health policy, enhance patient choice and optimise healthcare delivery.

## Methods

A protocol for this review was registered on the PROSPERO international prospective register of systematic reviews (registration number CRD42021269254), and the following approaches to systematically reviewing the available guidelines were implemented.

### Literature search

A search strategy was developed in order to identify relevant guidelines published between January 2000 and May 2020, for treatment of obesity in children and adolescents by searching electronic databases (MEDLINE, EMBASE, and CINAHL), as well as searches of reference lists. Searches were limited to articles published no earlier than the year 2000, from which time childhood obesity prevalence accelerated in many regions (1), and due to the increased volume in childhood obesity intervention literature. Further, treatment guidelines older than 2000 were deemed likely to be outdated. Local health professionals specialising in paediatric weight management were consulted and asked to check included guidelines to ensure none were missed to their knowledge. Google searches for grey literature were also undertaken. An example of the search strategy can be found in the supporting material.

#### Inclusion/exclusion criteria

Inclusion criteria for this study entailed:

• clinical practice guidelines (as defined by the National Academy of Medicine) for the treatment of obesity, published in English, if they addressed treatment in children and adolescents aged 0–17 years.
• guidelines from specific disciplines of healthcare practice (e.g., nutrition/dietetics) were included if treatment of obesity in children was the primary objective.

Guidelines were not eligible for inclusion if they:

• discussed childhood obesity, but did not present specific recommendations;
• did not define a systematic search for evidence;
• addressed obesity treatment in populations with other primary diagnoses such as type 2 diabetes mellitus (T2DM);
• focussed on prevention rather than treatment of childhood obesity;
• were published before 2000;
• were written in non-English language;
• addressed populations >18 years;
• were delivered outside healthcare settings.
• did not reach the pre-specified cut off score for quality according to the AGREE II tool (described below)

#### Quality assessment

The Appraisal of Guidelines for Research & Evaluation II (AGREE II) tool (45) was used to assess the methodological quality of each clinical guideline. The AGREE II scale consists of 23 items in six domains: (i) scope and purpose, (ii) stakeholder involvement, (iii) rigour of development, (iv) clarity of presentation, (v) applicability, and (vi) editorial independence. Each item was scored from 1 (strongly disagree) to 7 (strongly agree), and domain scores were calculated by summing the scores of the individual items in a domain and by scaling the total as a percentage of the maximum possible score for that domain. This scoring protocol was implemented using the “My Agree” tool,^1^ where scores of 1–3 indicate that the guidelines are not recommended, 4–5 to indicate “recommended with modifications,” and scores of 6 and 7 to indicate that the guidelines were recommended. The AGREE II consortium has not set specific cut-off scores to differentiate between high and low-quality guidelines, therefore, we designated a threshold that a guideline of acceptable quality required a score of at least 60% for “rigour of development” (domain 3) as well as 60% in at least two other domains. This quality threshold was based on cut-off scores reported in a previous review (46). Each guideline was reviewed by two members of the review team, and a third member was consulted where there was disagreement in the eligibility.

#### Data extraction

The following data were collected from the included guidelines: publication details, guideline objective, methodology used to compile guideline, setting of care, and general recommendations. We also extracted detailed components of recommendations made within the following treatment foci for children and adolescents: defining obesity, diagnosis, assessment, behaviour change techniques; nutritional interventions; interventions for improving physical activity and/or sedentary behaviour, sleep, and screen time; management of psycho-social factors; pharmacological approaches; surgical approaches; contact time provided by practitioners or length of intervention; professional characteristics of those delivering treatment (qualifications, professional accreditation, training); use of digital media in treatment; level of parent/carer/family involvement; age-specific recommendations; guidance on communication; and social service and child welfare considerations.

To ensure consistency and quality assurance, the abstract, title and full text screening (CW/NA/GOM/LT) of the retrieved records, as well as the AGREE II appraisal (CW/NA/LC/LMc) and data extraction (CW/NA/LT), were each independently completed by two review authors.

## Results

### Literature

**FIGURE 1:**
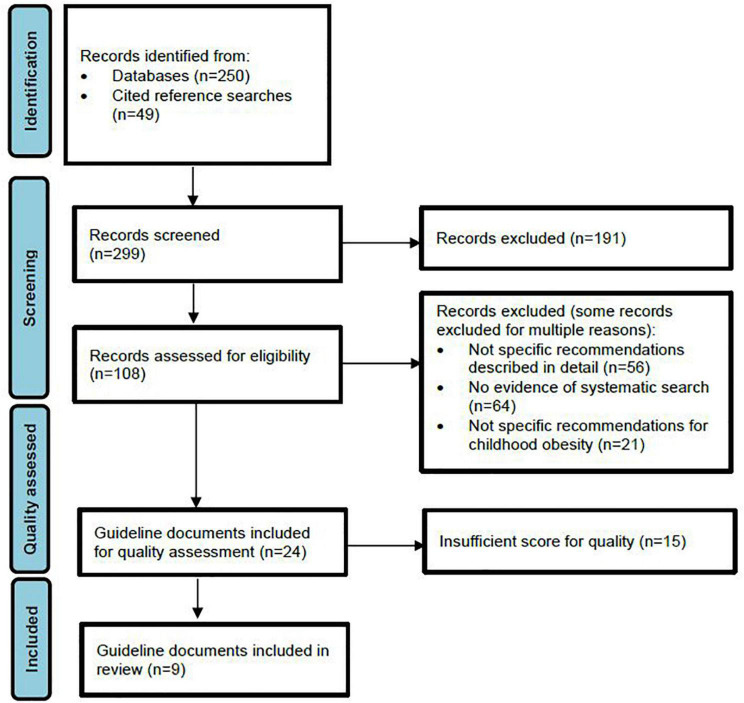
PRISMA flow diagram.

Results of the literature search and selection process are illustrated in Figure 1. Initial searches using databases (MEDLINE, CINAHL, and EMBASE) yielded 250 guidelines, while grey literature and reference list searches identified a further 49 guidelines. Of these, title and abstract screening identified 108 guidelines suitable for full text review, with 24 of these meeting our criteria for inclusion taken forward for analysis using the AGREE II tool. Of the 83 guidelines that were not selected for AGREE II evaluation, 71% failed to present detailed, specific recommendations, 82% were excluded due to absence of detailed systematic search procedures, 27% were exclusively adult recommendations and 45% did not focus on the treatment of childhood obesity. Many guidelines were excluded by a combination of these criteria. Application of the AGREE II tool yielded nine guidelines of sufficient quality for inclusion in the final review (Table 1) (47–55). The list of guidelines assessed for inclusion can be seen in the Supplementary Table 1.

**TABLE 1:** Clinical practice guidelines deemed to be high quality and included in the review.

Author/Organisation	Title	Country of origin	Overall AGREE II score
Academy of Nutrition and Dietetics (AND) (47)	Paediatric Weight Management Guideline	USA	4.0
American Psychological Association (APA) (48)	Clinical Practice Guideline for multicomponent behavioural treatment of obesity and overweight in children and adolescents	USA	6.0
Australian National Health and Medical Research Council (AUST) (49)	Clinical practice guidelines for the management of overweight and obesity in adults, adolescents and children in Australia	Australia	6.5
Spanish Ministry for Health (SPAIN) (50)	Working Group of the Guideline for the Prevention and Treatment of Childhood and Juvenile Obesity. Clinical practice guideline for the prevention and treatment of childhood and juvenile obesity	Spain	5.5
Scottish Intercollegiate Guideline Network (SIGN) (51)	Management of Obesity – a national clinical guideline	UK	6.5
National Institute for Clinical Excellence – UK (NICE) (52)	Obesity: identification, assessment and management - Clinical guideline [CG189]	UK	6.5
Canadian Task Force on Preventive Health Care (CAN) (53)	Recommendations for growth monitoring, and prevention and management of overweight and obesity in children and youth in primary care.	Canada	4.0
Styne et al. Endocrine Society (STYNE) (54)	Obesity—Assessment, Treatment, and Prevention	USA	6.0
World Health Organisation (WHO) (55)	Guideline: assessing and managing children at primary health-care facilities to prevent overweight and obesity in the context of the double burden of malnutrition.	Global	6.5

#### Quality assessment

The overall scores for the six AGREE II tool domains and overall assessment scores are shown in Supplementary Table 2. There were nine guidelines determined to be of sufficient quality for inclusion based on the AGREE II criteria, scoring over 60% in domain 3, “Rigour of development” and more than 60% in any other two domains. The mean score for applicability was 39%, with many guidelines scoring poorly on this parameter, seven scored less than 20%. Mean scores for “clarity of presentation” was 60% and four guidelines [AUST (49), NICE (52), SIGN (51) and Yi (56)] scored greater than 90%. The domain with the lowest mean score (44%) was “stakeholder involvement” with six guidelines scoring less than 20%. Two guidelines scored highly for “stakeholder involvement,” NICE (52) (92%) and AUST (49) scored maximum for this domain. The mean score for “editorial independence” and “scope and purpose” were 51% and 67%, respectively. “Rigour of development” was an important domain in this study, which assessed the quality of the methods used to process the evidence that supported the recommendations. The mean score for this domain was 51% with NICE (52), SIGN (51), AUST (49), APA (48), STYNE (54), and WHO Primary Care (55) all scoring over 90%. Overall, 9 guidelines met the criteria for further analysis (see Table 1) based on the scores allocated using the AGREE II tool.

#### Population considered

Guidelines (or sections of guidelines aimed specifically at treating obesity) predominantly considered populations of children aged 0–18 years, whilst two guidelines (CAN and APA) considered 2–18 years. The WHO guidelines included in the review were aimed at treatment of under-fives only. The American Dietetic Association’s guidelines through the Academy of Nutrition and Dietetics (AND) focused on treatment in children six years and older (47). The APA highlighted evidence supporting interventions at the earliest age possible.

#### Defining obesity

All guidelines recommended use of BMI percentiles or standardised BMI scores based on various reference groups (57–63) for considering a child to be overweight or to have obesity. Overweight and obesity were included as part of the treatment guidelines for the APA, AND, CAN, NICE, AUST, and WHO guidelines while all others considered only obesity. Table 2 summarises the definitions of obesity used in each included guideline.

**TABLE 2:** Definition of obesity for children used in each included guideline.

Guideline	Reference group/Growth chart	Obesity cut-off
AND	International Obesity Task Force (IOTF 2000) (57)	≥95th percentile
APA	Center for Disease Control (CDC) reference group (58)	≥95th percentile
CAN	World Health Organization (WHO) growth charts for Canada (59)	2–5 years: ≥99.9th percentile 6–17 years: ≥97th percentile
AUST	CDC (58) or WHO charts (60)	>95th percentile (US–CDC) >97th percentile (WHO)
STYNE	CDC (58) or WHO charts (60)	≥95th percentile
SPAIN	Growth curves and tables of Hernández et al. (61)	≥97th percentile
WHO	WHO Integrated Management of Childhood Illness (IMCI) references (62)	>3 standard deviations (SD) of the median
NICE	1990 UK reference data (UK90) (63)	Not defined: consideration for tailored intervention recommended ≥91st percentile; assessment of co-morbidity recommended ≥98^th^ percentile
SIGN	UK90 (63)	≥98th percentile

#### Setting

Six of the nine included guidelines (AND, APA, AUST, SPAIN, SIGN, and WHO) discussed the target setting of their guidance for delivery of treatment. AND, APA and SPAIN recommended flexibility in delivery for the intervention itself, which although clinically designed within a health service and healthcare professional (HCP) delivered, could take place inside or outside the clinical setting. The APA elaborated that with insufficient evidence to recommend an optimal setting, the aim should be to reduce access barriers by choosing suitable venues to increase accessibility such as community, medical or faith-based centres. The AUST guidelines, whilst focused on delivery for HCP in general practice, also found insufficient evidence for guidance on the setting of the intervention itself, as did the AND. The WHO guidelines are aimed at recommendations for delivery of primary care in low- and medium- resourced settings broadly. SIGN specified that their guidance were for use within UK primary, secondary and tertiary health services generally, and highlight that no evidence was found for delivery of weight management in residential or camp-style treatments.

Only one guideline (AND) referred to the resources needed for the recommended multidisciplinary team (MDT) weight management programme, stating that the costs and resources required depended on the staffing, setting, format of sessions, duration/frequency of the intervention, and insurance coverage/reimbursement. The CAN guidance explicitly stated that the resource needs for provision of treatment were not reviewed.

#### Profession and training requirements for delivery

The APA and AND guidelines, although specific to registered dietitian nutritionists (AND) and psychologists (APA), recommended care as part of a MDT for childhood obesity treatment. The AUST guidelines were predominantly aimed at general practitioners and those working in primary care. No other guidelines were aimed at any one specific profession, though many specified the need for a specialised MDT.

Regarding training requirements, most of the guidelines (7/9) recommended that healthcare professionals caring for children/adolescents with obesity have specific training/expertise in paediatric obesity management (APA, AND, CAN, NICE, SIGN, SPAIN, and WHO). Specific recommendations for training requirements included weight management in children with intellectual disability (NICE), weight bias and stigma (APA), behaviour change counselling (SIGN), paediatric training and experience (SPAIN), and growth measurement training (WHO). The CAN and NICE guidelines recommended a specialised team for obesity treatment. No other explicit training recommendations were made, except whereby surgical or pharmacotherapy was recommended and these were only to be under appropriate speciality supervision. Notably also the AND guidance was aimed specifically at registered dietitians and nutritionists and therefore assumed such expertise in its advice on medical nutrition therapy.

Two guidelines recommended that the effectiveness of training for HCPs in childhood obesity should be investigated in order to identify the approach that is most likely to improve child health outcomes (AUST, SIGN).

#### Obesity diagnosis and related assessments

The APA guidelines explicitly stated that assessment of childhood obesity was not addressed in their guidelines, whilst the CAN recommendations did not provide guidance for assessment beyond growth monitoring. All others provided some level of recommendation for assessment. These are summarised in categories in Table 3.

**TABLE 3:** Summary of specified assessment categories in guideline recommendations.

Guideline	Factors recommended for assessment of childhood obesity											
	Family history/ Risk factors	Eating behaviour	Sedentary behaviour	Physical activity	Resting metabolic rate	Family climate/Home environment	Full clinical history	Underlying conditions and/or comorbidities	Mental health/ Psychological factors	Sleep	Schooling	Use of obesogenic medication
Academy of Nutrition and Dietetics (AND) (47)		x				x						
National Institute for Clinical Excellence (NICE) (52)	x	x	x	x		x	x	x	x			
Scottish Intercollegiate Guideline Network (SIGN) (51)							x	x				
Spanish Ministry for Health (SPAIN) (50)						x	x	x	x			
Australian National Health and Medical Research Council (AUST) (49)		x	x	x		x	x	x	x	x	x	
Styne et al. Endocrine Society (STYNE) (54)	x	x	x	x		x	x	x	x			
World Health Organisation (WHO) (55)							x	x				

STYNE also made recommendations against testing in certain cases, including routine laboratory evaluations for endocrine aetiologies of paediatric obesity unless the patient’s stature and/or height velocity are attenuated (assessed in relationship to genetic/familial potential and pubertal stage). Genetic testing was only recommended in patients with extreme early onset obesity (before five years of age) and that have clinical features of genetic obesity syndromes (in particular extreme hyperphagia) and/or a family history of extreme obesity. The authors also recommended against measuring insulin concentrations when evaluating children or adolescents for obesity.

Assessment of psychosocial or emotional distress, including the incidence of bullying, teasing and low self-esteem, was recommended in four guidelines (AUST, NICE, SIGN, and STYNE). Finally, NICE highlighted that HCPs should be aware that child abuse may contribute to, co-exist with or cause obesity, and refer to NICE guidance on child maltreatment. None of the guidelines recommended assessing previous prescription of obesogenic medications.

#### Monitoring and onward referral

Four of the nine guidelines included reference to monitoring growth in the long term (AUST, STYNE, SIGN, and WHO). STYNE and SIGN recommended plotting the child/adolescent’s growth at least annually, to reinforce weight management (SIGN), and as part of routine well-child or sick visits (STYNE). WHO similarly recommended follow-up in primary care if resources were available, but did not specify a frequency. The AUST guidelines recommended measurements (for BMI, health behaviours, and co-morbidities) every three months or more frequently during treatment, and “regularly” in the long term. It was recommended that these measures in addition to clinical judgement inform any decision to refer onward, while the role of the primary healthcare provider remains to support lifestyle change (AUST).

Referral to structured weight management programmes or specialised services were recommended for those in primary care who are unable to deliver such interventions (CAN) or who are unable to assess obesity or manage complications (WHO). The AND guideline further recommended registered dietitians and nutritionists be aware of community resources for which to signpost. The AUST, NICE, and SIGN guidance recommended referral to hospital or paediatric services for:

(i) those >95th percentile on US-CDC growth charts or the 97th percentile on WHO growth standard charts (AUST);
(ii) (those under 2 years, above the 97th percentile on WHO growth charts and gaining weight rapidly (AUST) or those at ≥99.6th percentile (SIGN, UK90 charts);
(iii) those who may have serious co-morbidities requiring weight management (NICE, AUST, and SIGN);
(iv) those for whom an underlying medical, endocrine or development issue is suspected (AUST, SIGN), or those with complex needs (e.g., disabilities) (NICE).

For post-pubertal patients with severe obesity and co-morbidities, intensive interventions through specialist clinics should be considered where lifestyle modifications have not been successful and the potential benefit outweighs risks (AUST). Additional specialty referral considerations were recommended in certain circumstances such as where there were new co-morbidities or symptoms, signs of psychological distress (refer to psychology), signs of disordered eating (refer to dietetics), or parenting/family problems (refer for parenting assistance) (AUST). For most children managed in the community, SIGN surmised, would likely have “simple” obesity and no complications. SPAIN guidance recommended endocrine referrals for those with underlying disorders associated with obesity, and mental health referrals for psychiatric disorders. NICE highlighted the need for arrangements for transitional care for those moving to adult services, while AUST also recommended having a HCP responsible for the transition to adult care and having a service provider accept responsibility for active case management once the adolescent/young adult has left the paediatric/adolescent service. The APA highlighted the benefits of treatment within integrated healthcare systems to include improved adherence to programmes, fewer hospitalisations, and improvements in patient outcomes.

### Lifestyle modification-focussed treatment

#### Outcome goal

NICE guidelines expressed the importance of considering the child and families’ own preferences in deciding the outcome goals of treatment. NICE specified the need for realistic goals which may focus on outcomes other than weight loss, such as increased physical activity and healthy dietary intake. They also advised a steady decline in BMI in order to preserve lean mass, as did STYNE.

Weight stabilisation/maintenance was considered a widely appropriate treatment goal by SIGN, STYNE, AUST, and SPAIN. The APA clarified that change in BMI/BMI z-score only were assessed in their literature review and therefore additional goals were not considered. One guideline (CAN) stated that management should focus on reducing BMI and secondary outcomes including total cholesterol, triglycerides, high-density-lipoprotein (HDL) cholesterol, low-density-lipoprotein (LDL) cholesterol, two-hour fasting blood glucose, systolic blood pressure, diastolic blood pressure, overall quality of life and physical fitness.

SPAIN specified an exception to a goal of maintaining constant weight whereby a child is >99th percentile or presents with comorbidities. In such cases, gradual weight loss was recommended, not exceeding 400 g per month in children aged 2–5 and not exceeding 800 g a week in children and adolescents aged 6–18. Consideration of tracking waist circumference as an indirect estimator of visceral fat content was also recommended by SPAIN. SIGN made similar recommendations, whereby if children presents with a BMI ≥99.6th centile, a gradual weight loss to a maximum of 0.5–1.0 kg per month is acceptable. Importantly, SIGN noted a gap in understanding for the extent to which change in BMI or other parameters would affect obesity-related co-morbidities. AUST guidelines suggested a focus on family behaviours instead of the individual child’s weight was preferable.

#### Treatment format and duration

Four guidelines stated that treatment should be tailored to the preferences, cultural values, and needs of the family (AND, CAN, NICE, and SIGN), and should only be undertaken with families who are ready and willing to participate (SIGN). The CAN guideline also recommends communicating clearly to parents that there is limited evidence for the effectiveness of programmes, to help families make decisions.

Four guidelines (AND, AUST, CAN, and NICE) emphasised the importance of having a MDT of health professionals. Five guidelines explicitly specified that treatment should encompass multi-component interventions (AUST, NICE, APA, SIGN, and AND), although each guideline contained various multiple components. A group treatment format was recommended by the CAN, while the AND reported no consensus on group versus one-to-one treatment delivery. The AND reported that a group format with family involvement had evidence for long term success, while individual sessions with or without family involvement had evidence for short term success with mixed findings in the longer term.

The APA reported insufficient evidence to support any particular format or mode of delivery, but suggested that technology and mobile–health (mHealth) approaches may provide additional solutions to increasing access to treatment. Both APA and AUST guidelines reported a need for evidence for the role of technology in augmenting care.

Two of the guidelines (APA, AUST) included a broad recommendation of frequent contact between the child/adolescent and family and the MDT. Another (CAN) recommended that interventions should include several sessions, adding that the interventions reviewed occurred over weeks to years. The AND and SIGN guidelines were more specific stating a time frame of at least 6 months duration for interventions, while the APA recommended a minimum of 26 contact hours and commenced at the youngest age possible. The AND reported a lack of evidence to specify a number of contact hours. For behavioural counselling, SIGN recommended eight sessions over six months. These guidelines also stated that “intensive and longstanding” weight management programmes were most effective in young people. AUST guidelines stated that the frequency of treatment sessions should be balanced with burden on the family. The APA also reported observing no difference in attrition rates between high intensity and low intensity interventions, however it is important to note that they excluded studies with very high attrition rates.

Having a tailored or personalised approach is stated in many (APA, AUST, NICE, SPAIN, and STYNE) of these guidelines, while STYNE and AND added that this should entail intensive, age-appropriate, culturally sensitive, family-centered lifestyle modifications. Parental/family participation in treatment was widely encouraged (AND, SPAIN, CAN, NICE, AUST, STYNE, and SIGN) especially for children under 12 years whereby parents/families can be role models and agents of change (NICE, AUST, WHO, and SIGN). The APA notably reported inconclusive evidence as to whether family or individual sessions were more efficacious, highlighting room for a tailored approach.

#### Communication style

AUST guidance recommended that communication focus on benefits of a healthy lifestyle for the whole family rather than the weight of the child undergoing treatment, and this was supported by the APA who stressed that this was a population (children and parents) already subject to increased stigma who should be met with non-judgemental and non-stigmatising care. NICE recommended accounting for specific communication needs (for example because of learning disabilities, physical disabilities or cognitive impairments due to neurological conditions) and clear, appropriate communication focusing on praise at every opportunity. The AND recommended considering literacy to ensure appropriateness of resources.

#### Nutritional intervention

All nine guidelines recommended some level of nutrition support within childhood obesity treatment, and the level of guidance detailed varied substantially. Five guidelines (AND, APA, AUST, SPAIN, and STYNE) stated that the nutritional intervention should be delivered by a member of the specialist MDT.

Notably, the AND guidelines did not review all components of their previous 2007 guideline (64), which included both prevention and treatment recommendations for obesity with detailed nutrition intervention recommendations, but did not meet the eligibility criteria for this review. In their 2015 guideline (included in this review), they recommended advising a reduction in the frequency of fast-food intake to less than twice a week.

SIGN stated that there was no evidence to suggest any particular dietary or macronutrient manipulation. The AUST recommended that very low energy diets may produce short term rapid weight loss but noted lack of long-term follow up data, and the need for continued weight management after such an intensive intervention.

Four guidelines (NICE, SPAIN, STYNE, and AUST) recommended advice based on local health department guidelines while considering the food environment and needs of the family. AUST additionally recommended focussing on recognising internal food cues and promoting eating as a family away from screens, with a general approach of reducing energy intake.

The WHO guidelines recommended nutrition counselling for parents while acknowledging this as broad advice which may be insufficient alone and may need to be context specific. Detailed nutrition guidelines were out of scope but recommended to be in line with local guidance and food availability.

NICE and SPAIN, guidance recommended against use of restrictive, unbalanced diets, while NICE further recommended dietary improvement for overall health, regardless of weight. SIGN highlighted limited evidence for effectiveness of reduced energy intake. The CAN guidelines recommended against very low kcal diets for preadolescents. SIGN further recommended against any particular dietary or macronutrient manipulation due to insufficient evidence.

Three guidelines (AUST, SIGN, and STYNE) specifically mentioned portion sizes. One guideline (APA) advised on matching caloric intake to meet energy expenditure through energy balance behaviours. Two guidelines (AND, STYNE) included recommendations around fast food consumption and two others (SIGN, STYNE) on reducing consumption of foods high in fat and added sugar. The AUST guidelines encourage promotion of healthy comfort behaviours that help to discourage using food to regulate emotions.

#### Interventions to improve physical activity

The inclusion of physical activity as part of treatment for childhood obesity was recommended in all of the included guidelines, but the level of detail provided within the guidelines varied.

For young children (preschool), the WHO recommend counselling of caregivers of children under five years in primary care. The WHO also noted that there is limited evidence for the effectiveness of counselling, however, as well as the format of interventions.

For children and adolescents more generally, NICE, AUST, and SPAIN recommended that physical activity should be suited to the child’s age, interests and ability in order to encourage and maintain engagement. NICE added the benefits of encouraging physical activity regardless of change in weight, while AUST guidance encouraged parents as role models and making use of local facilities and opportunities to engage in sports. Three guidelines recommended that children generally needed at least 60 min of moderate to vigorous activity a day (NICE, SIGN, SPAIN), one of which (NICE) suggested that the 60 min of activity could be broken up throughout the day into several sessions lasting 10 min or longer. They also recommended regular structured activities, while adding that for children who are already above a healthy weight, more than 60 min per day may be necessary. STYNE suggested prescription and support for moderate to vigorous activity levels, which should be gradually increased from 20 to 60 min a day, while SIGN noted the lack of clarity in evidence for amount and intensity of exercise.

It was suggested by one guideline that children should be given the opportunity and support to do more exercise in a structured environment (NICE). Some guidelines recommended that children should be encouraged and supported to do more spontaneous exercise in their daily life such as active play, walking and using stairs (SIGN, NICE, and SPAIN), while the CAN guidelines similarly recommended broad strategies which include counselling, support, and environmental adaptations.

#### Sedentary behaviour, screen time, and sleep hygiene

Five of the nine guidelines specifically recommended encouraging families to decrease sedentary activities such as sitting and screen time (AUST, NICE, SIGN, SPAIN, and STYNE). Three guidelines suggested that non-educational screen time such as watching television and playing computer games should be limited to a maximum of 1.5–2 h per day (SIGN, SPAIN, and STYNE). The SIGN guidelines noted that this was based on expert opinion, and the mechanism for reducing screen time is unclear, but it was likely to increase physical activity.

The guidelines for SPAIN also recommended removal of televisions and consoles from bedrooms.

The CAN guidelines reported no available evidence for interventions to improve sleep. No other clinical guideline referred to sleep hygiene or improvements in sleep in their recommendation or evidence syntheses.

#### Behaviour change strategies

Most guidelines recommended the use of behaviour change strategies, though neither WHO nor STYNE mentioned these explicitly. SPAIN and AUST recommended broadly that these be included as part of a psychological intervention component, and CAN similarly recommended general behaviour change counselling, adding that it is most effective when provided by a trained professional as part of a multi-component intervention. The APA stated that there was room for flexibility given the insufficient body of evidence to recommend any one behaviour change strategy. NICE and CAN emphasised that treatment delivery to be undertaken by an appropriately trained professional.

Specific behaviour change strategies commonly recommended as part of treatment for obesity included goal setting and self-monitoring (NICE, AUST, and SIGN), stimulus control (NICE, SIGN), and problem-solving (AUST). The AUST guidance also discussed evidence for incentives in the short term, and outlined the use of tools such as the SMART (specific, measurable, achievable, realistic, and timely) tool to assist families with goal setting. NICE highlighted that decisions such as setting goals and actions need to be made with the child/adolescent with overweight/obesity and the family.

#### Psycho-social support/therapy

Psycho-social risk factors were directly or indirectly mentioned by eight of the nine guidelines, while those that recommended interventions focussed primarily on psychological interventions (SPAIN, AUST, STYNE, and APA). The CAN guidelines specified that no evidence was identified for the effectiveness of mental health interventions for obesity treatment in childhood, and the WHO, SIGN, and AND guidelines did not make any recommendations specific to provision of psychosocial support. The SIGN guidelines did highlight evidence for improvement in self-esteem and quality of life among children who participated in multi-component lifestyle programmes.

For specific psychological support, the APA guidelines focussed heavily on this aspect of treatment due to its focus specifically for psychology professionals, whilst considering the necessity for psychological support in the context of a multi-component intervention. In particular the APA guideline referred to reducing stigma within families. The SPAIN guidelines specifically recommended forms of behavioural therapy as part of treatment, including stress reduction strategies, either individually or as part of a group multi-component programme. NICE recommended advice on self-care within family-orientated intervention.

The AUST guidelines recommended addressing disordered eating, poor body image, depression and anxiety, and weight-related bullying where these are identified during assessment, while STYNE (54) also recommended that psychosocial support and counselling by a psychologist be provided where issues are identified during assessment.

### Pharmacotherapy

Pharmacological interventions were discussed in all but two (WHO, APA) of the nine guidelines. It should be noted that the evidence has evolved rapidly regarding use of pharmacotherapy for obesity (65, 66), and many of the studies that informed these guidelines are likely now out of date.

The seven other guidelines reviewed only recommended pharmacological treatment of obesity in exceptional cases for children with severe obesity or comorbidity (CAN, NICE, SIGN, SPAIN, and STYNE), in adolescents (CAN, NICE, SIGN, and SPAIN), following failure of other interventions (AUST, NICE, SPAIN, and STYNE). NICE added that pharmacological treatment in those <12 years may be needed in exceptional circumstances. Pharmacological treatment of child and adolescent obesity should be supervised by a specialist MDT that maintains regular assessment of progress (AUST, NICE, SPAIN, STYNE, and SIGN) and with careful monitoring of side effects (SIGN).

Orlistat was considered by five of the nine guidelines (CAN, NICE, SIGN, SPAIN, and STYNE) to be the only current pharmaceutical option for treatment of obesity in children and adolescents (where specific guidelines around use may vary from country to country following national regulations). One guideline considered the use of metformin in children/adolescents with obesity (SPAIN). Agents that have been withdrawn since the publication of the guidelines (sibutramine, rimonabant) were not considered any further in this review. Two guidelines recommended that Orlistat treatment be accompanied by supplementation of fat-soluble vitamins (NICE, SPAIN), while the AND recommended the involvement of registered dietitians in pharmacotherapy. The clinical threshold at which Orlistat use might be considered was quantitatively defined by one guideline (SIGN) as when a child had obesity (BMI ≥ 99.6th centile on UK 1990 reference range for age and sex) and comorbidities, or very severe obesity (defined as BMI ≥ 3.5 SD above the mean of the 1990 UK reference range).

### Surgical procedures

Six of the nine included guidelines addressed bariatric surgery. The guidelines reviewed either made no recommendation (APA, WHO) or recommended that surgical procedures for treatment of obesity in adolescents are offered only in cases of extreme obesity or comorbidities (AUST, CAN, NICE, SIGN, SPAIN, and STYNE), and when other interventions have been unsuccessful (AUST, STYNE, SPAIN). Guidelines that did consider the use of surgical procedures in children/adolescents with obesity were in agreement on the necessity that this intervention is delivered by a highly specialised MDT (AUST, NICE, SIGN, SPAIN, and STYNE). Two guidelines recommended that the infrastructure for patient care (surgical and rehabilitation facilities and healthcare professionals capable of long-term follow-up) are in place before surgical procedures are considered (NICE, STYNE). Most guidelines recommended that surgical procedures are considered only in adolescents that have reached physiological (and psychological, SPAIN) maturity (AUST, NICE, SIGN, and SPAIN). STYNE further stipulated recommending bariatric surgery only if the patient could adhere to healthy dietary and activity habits.

All guidelines that considered surgical procedures acknowledged the lack of randomised clinical trials to support recommendation of surgical treatments in children or adolescents with obesity (AUST, CAN, NICE, SIGN, and SPAIN) and four guidelines recommended future research on the long-term outcomes of surgery in children/adolescents with obesity (AUST, CAN, NICE, and SPAIN). Three guidelines recommended communicating the risks, and the commitment to long-term follow up of surgical procedures for obesity to patients and their families (SIGN, SPAIN, and NICE). NICE recommended that all young people have a full medical evaluation and comprehensive psychological, educational, family and social assessment before undergoing bariatric surgery. Four guidelines presented clinical thresholds at which surgical procedures might be considered:

• laparoscopic adjustable gastric banding with BMI >40 kg/m^2^ or >35 kg/m^2^ with obesity-related complications (AUST, STYNE) which are significant and extreme (STYNE);
• bariatric surgery (unspecified) considered with BMI ≥3.5 SD above the mean on 1990 UK charts (SIGN);
• bariatric surgery (unspecified) considered with BMI ≥40 kg/m^2^ and severe comorbidity, or BMI ≥50 kg/m^2^ (SPAIN).

NICE further recommended pre- and regular post-operative assessments including dietetic assessment including eating disorder risk, information regarding plastic surgery if needed, ongoing psychological support, and access to suitable bariatric equipment (e.g., hoists, suitable seating), with staff trained to use them. The SPAIN guidelines further specified the possible need for lifelong follow up, while AUST highlighted a lack of evidence for beneficial or harmful consequences more than 12 months after surgery, as studies did not follow up beyond this time.

The SPAIN guidance additionally reported that no information was identified on the efficacy of intragastric balloons in the treatment of adolescents with obesity.

### Risks and complications of obesity and its treatment

#### Management of complications of obesity

With regards to the management of complication of obesity, NICE recommended this be initiated when co-morbidities are identified, rather than after weight loss has occurred. AUST recommended that physical and mental health co-morbidities are assessed and monitored throughout the intervention and follow up, adding that onward specialist referral or modification of treatment may also be needed, whilst the WHO made similar recommendations highlighting the need for a weight management plan that included dealing with complications, with referral as appropriate, based on capacity at the centre. The SIGN guidelines noted that serious obesity-related co-morbidities may need treatment, which might also be enhanced by weight management. In such cases, weight loss rather than maintenance might need to be the aim of treatment. SIGN guidance also recommended screening for obesity in addition to short stature for age as a potential indicator for an underlying medical condition.

#### Adverse events in obesity treatment

Seven of the included guidelines referred at some point to the management of risks associated with treatment or aspects of treatment for obesity (AND, APA, AUST, CAN, SPAIN, SIGN, and NICE). The CAN and SIGN guidelines highlight the dearth of evidence on adverse events from treatment itself, and of the long-term benefits or harms of treatment programmes. The AND suggested that school-based interventions may risk stigmatisation. SPAIN recommended monitoring for signs of, or risk factors for eating disorders. The APA suggested that weight management risks included family conflict arising or development of psychological issues for the child or adolescent related to the “success or failure” of the intervention. Of the studies reviewed by the APA, it was reported that few assessed psychological wellbeing as a treatment outcome, but some (*n* = 11) assessed quality of life, one of which reported a possible negative impact of the intervention.

The AND advised that exercise interventions risk injury and should only be undertaken after medical clearance, while AUST guidance advised that vigorous activity interventions be balanced with potential adverse effects on growth.

Physical risks highlighted also included nutrient deficiency, which SIGN and NICE referred to specifically in relation to pharmacological treatment for at risk groups such as adolescents. According to AUST, Orlistat was noted to increase the risk of adverse events. SPAIN advised that pharmacotherapy is not approved for children, and so consent must be sought and thorough explanation of side effects explained to parents prior to use.

It was emphasised that the use of pharmacotherapy for obesity may increase the risk of harm and side effects (CAN), and the long-term tolerability is not well researched, with close monitoring vital (AND, SPAIN, NICE, SIGN, and STYNE).

For surgery, the AUST recommendations highlight that risks varied by procedure but severe complications arose in around 5% of cases, with limited data on potential harms or benefits beyond 12 months. SPAIN guidelines highlighted the risk of micronutrient deficiencies after bariatric surgery and also recommended supplementation if required.

#### Burden on families

The CAN, AUST, AND and APA guidelines acknowledged the burden on families for attending weight management programmes, and the need to be mindful of this. The CAN guidance added that this may result in families declining to participate, while the AND and APA noted potential burdens of treatment to include absenteeism from school or work, childcare needs, transport, costs, and the AND added that lack of insurance coverage may also be a barrier to participation. The APA guidance further emphasised the potential burden on families of accessing safe physical activity and healthy food, having one or more parent available to attend treatment, and the possibility that the child may suffer academically. They recommended addressing perceived barriers during treatment and noting that socioeconomic status may be a factor, however, these recommendations were based on anecdotal evidence. The AND also recommended assessing socioeconomic status, and added that if economic issues were perceived to be a concern, a referral to social services should be considered.

**FIGURE 2:**
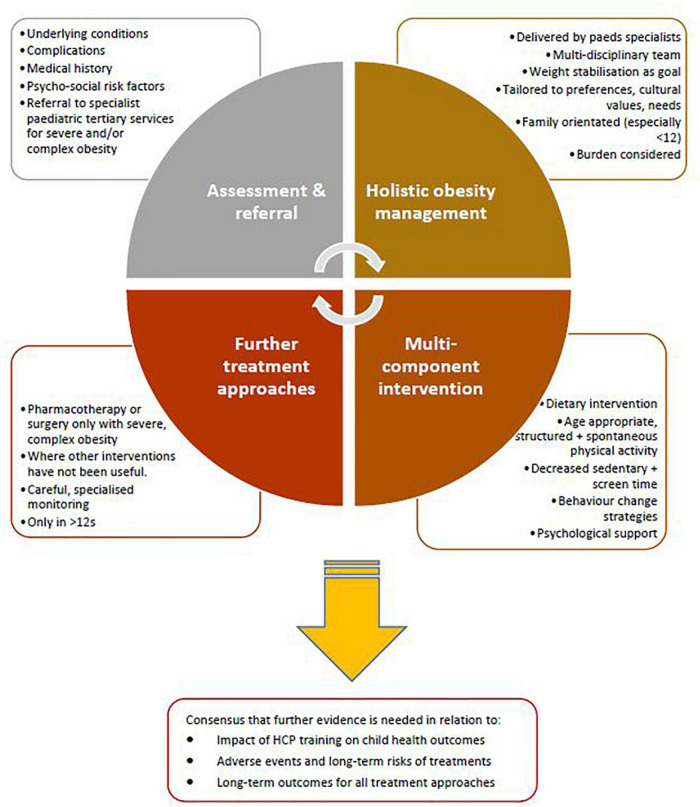
Approaches to paediatric obesity management for which there was general agreement across included guidelines from high income countries.

The approaches to treatment of obesity for which there was general consensus across the included guidelines are summarised in Figure 2.

## Discussion

### Principal findings

We systematically reviewed existing international clinical practice guidelines for the treatment of obesity in children and adolescents.

We assessed the standard and rigour of guideline documents in order to include those of the highest quality. In the narrative synthesis we compared recommendations to aid decision making by health professionals, healthcare managers or health policy makers.

The clinical guidelines included in our review came from five notably high-income countries (Australia, Canada, Spain, UK, and USA), in addition to the WHO. Comparing different levels of quality accurately across guidelines was not possible as various grading scales were used; although the majority of guidelines [APA (48), AUST (49), CAN (53), STYNE (54), WHO (55)] used the GRADE method to assess the quality of recommendations. Some recommendations, particularly for surgery and pharmacotherapy, were based on low quality evidence or expert consensus. To translate guidelines into clinical practice effectively, it is essential that the evidence base is of high quality and that a carefully planned implementation approach is used (67).

The importance of education and training of HCPs in the treatment of child and adolescent obesity, and in particular for those applying surgical procedures or pharmacotherapy, was identified by most of the guidelines. Most guidelines proposed that the clinical recommendations they present could be used in HCP educational programmes. Training should be delivered to HCPs, social and educational staff, and all professionals involved in caring for children/adolescents with obesity particularly in primary care. The effectiveness of HCP education should be regularly assessed, and the educational material used should be non-discriminatory and regularly updated.

There were similarities in many of the recommendations across guidelines (figure 2). These included recommendations on family-based multi-disciplinary treatment, behavioural interventions and multi-component lifestyle interventions. Interventions that involve family lifestyle modifications are the most widely studied and often are the most effective at sustaining behaviour changes (68). However, this success greatly relies on the capacity of the parent/guardian to implement and continually encourage change (69) and positive family functioning, as poor family functioning including communication difficulties, conflict and behavioural control increases the likelihood of overweight and obesity (70, 71). This supports the need for obesity to be treated as a systemic disease, in order to both prevent its development in children, and prevent complications and deterioration of quality of life for those already living with the disease. Furthermore, the individual context of the child and family should be considered when determining the ‘success’ of treatment at an individual level and a thorough, holistic and child-centred clinical assessment guides the most appropriate treatment plan. For example, in a child with severe obesity who has withdrawn from school or playing with peers, a successful outcome of treatment might be related to quality of life and their participation in education and social activities rather than solely based on a reduction in body composition. For the adolescent with obesity and sleep apnoea, treatment success might encompass improved sleep quality and duration coupled with reduced fatigue even in the absence of changes in anthropometry. To date, systematic reviews and clinical trials have reported positive impacts of obesity treatment on body size, weight, body composition, cardiometabolic health, functional capacity, musculoskeletal pain, neuromusculoskeletal fitness (72–85) and cardiovascular health (41). Future research is needed to ensure that clinical trials and evaluation of obesity treatment encompass core outcome sets that include outcomes of interest to the child and family and not those solely of interest to health professionals, researchers or health managers. Although the role of the environment and health equity are not directly addressed within current treatment recommendations, they are vital components of the collective effort to stabilise obesity rates globally, and improve health outcomes for those living with obesity (86).

Apart from the APA guidelines, the guidelines included recommendations on nutritional intervention, physical activity promotion, behaviour change, pharmacological and surgical interventions. All guidelines were consistent in focussing treatment on behaviour changes that subsequently impact on weight status as opposed to directly targetting weight loss or having a direct weight loss focus. Maintaining weight or slowing further weight gain to encourage a steady decrease in BMI was an appropriate treatment target highlighted in the majority of the guidelines.

The extent and nature of obesity assessments, where they were recommended, varied greatly (Table 3), from assessment of lifestyle and behaviours, to medical history, psychosocial factors, or a combination of these. The Edmonton Obesity Staging System for Pediatrics (EOSS-P) (87) was not mentioned in any of the included guidelines, but provides a useful and evidence-based framework to stratify patients according to factors which go beyond anthropometric measures and account for metabolic, mechanical, mental health, and social milieu. The latter highlights the importance for guidelines to include assessing and managing weight bias from health professionals and internalised stigma or family stigma within interventions, particularly with evidence for association of stigma with adverse psychological effects such as depression and low self-esteem, in addition to avoidance of physical activity and disordered eating patterns (88–92). Training of healthcare professionals to reduce stigma within healthcare settings is also vital (93).

There was little consensus on the specifics of treatment format recommendations, including the number of contact hours, the age of initiation of treatment, group versus one-to-one sessions, or the mode of delivery, such as in-person, in-person plus mobile/digital health or via digital health alone. This hinders healthcare providers and decision makers in estimating the resources needed to provide best practice while ensuring value for money. Despite the recent increase in digital delivery especially since the COVID-19 pandemic (94), this is a relatively new field with a dearth of high-quality evidence for treatment effect or cost-effectiveness (95).

Most studies of childhood obesity interventions focus on BMI as the primary outcome measure. One guideline (APA) highlighted the necessity for research on interventions to detail other standardised outcome measures including psychosocial impact, self-efficacy and metabolic functioning. The quality of measuring and reporting outcomes in paediatric obesity trials has been criticised (82), and these findings suggest the need for a core outcome set for evaluations of interventions in this population. These should go beyond change in adiposity or body shape/size, and focus on meaningful improvements for the patient, including those which indicate participation in age-appropriate tasks and leisure activities, reduction of obesity-related complications, and management of co-morbidities. Improved differentiation is required around how co-morbid diagnoses (e.g., learning difficulty, psychological issue, or respiratory, rheumatology or neurology diagnoses) affect obesity development, progression or treatment, and how the direction of relationships should or could impact treatment decisions (83). The use of obesogenic pharmacotherapies to treat such conditions is an important consideration for clinical decision-making related to obesity treatment (96).

An important aim for an obesity treatment service should be to engage successfully with service users and process indicators of such success might include non-attendance rates, family engagement with the service or patient satisfaction. There were few recommendations within the included guidelines relating to measurement of process outcomes or attendance. The APA highlighted no difference in attrition rates based on intensity of interventions (number of contacts, length of sessions), but had notably excluded studies with very high attrition rates. This may have biased the findings on intensity and attrition, and highlights the need for clear consideration of studies reporting factors that affect engagement for development of future guidelines. Assessment of attrition rates in addition to investigating patterns of drop-out, trends in drop-out timing and characteristics, as well as qualitatively assessing reasons for non-completion, would provide important insight to help prevent attrition or target engagement efforts for future guidelines, especially as attrition is generally high for paediatric obesity interventions (97). The WHO chronic care model (98) is a useful framework to use in relation to the design, implementation and evaluation of paediatric services delivering treatment for obesity in children and adolescents. Furthermore, the ethics of treating childhood obesity given the potential side effects and risk of harm from treatment is an area which is often not discussed, especially for interventions aimed at lifestyle modifications, and many behaviour change trials fail to report whether or not adverse events are even monitored (99). Whilst for medication in particular, it was commonplace for guidance to be accompanied by statements related to potential side-effects or harms (AND, CAN, SPAIN, NICE, SIGN, and STYNE), only two guidelines (AND, SPAIN) highlighted the need to monitor for risks of eating disorders with nutrition interventions which is recommended in the literature (100). Healthcare professionals must furthermore, carefully consider the ethics and professionalism related to consequences of *non-treatment* of obesity. Given its recognition as a chronic, relapsing disease (7), and the well-documented lifelong risks of obesity-related complications (11–18), health professionals, health managers, insurers and policy makers alike, should carefully monitor and justify incidents where a child is not referred for treatment, has limited access to care or where infrastructural barriers to providing treatment exist. Ultimately, research in the field would be strengthened if intervention trials (101) and clinical services clearly described the process for classifying, monitoring and recording adverse events or unanticipated events during the phase of referring a child or adolescent for obesity treatment and subsequently when the intervention/treatment is accessed. In order to improve the quality of future guidelines, those involved in developing and reporting on clinical trials should use the TIDieR checklist (102).

Moreover, the area of consent, assent, and the personal autonomy of the child/adolescent warrants further discussion and guidance, particularly in relation to surgery or pharmacotherapy. As highlighted by Michaud and colleagues in advocating for the autonomy of adolescents with chronic conditions (103), The United Nations Convention on the Rights of Children (UN CRC), article 12 states that “State parties shall assure to the child who is capable of forming his or her own views the right to express those views freely in all matters affecting the child, the views of the child being given due weight in accordance with the age and maturity of the child” (104). Future clinical trials and guidelines should give consideration to the fine balance between parental consent, child assent and the transition from assent to consent when the adolescent becomes an adult in their local legal system.

Whilst the NICE and AUST guidelines referred to the need to assist and manage the transition to adult services for older adolescents, this is a period of risk (105), particularly in healthcare systems where adult obesity services are not accessible to all.

In terms of nutrition interventions, emphasis was placed on following healthy eating guidelines which differ across countries but generally included guidance on portion sizes and food groups that help achieve a balanced intake of nutrients for the growing child. Specific dietary advice was also missing due to the inclusion of only one dietetic guidelines from the American Dietetic Association (AND), which formed a partial update to a previous guideline which did not meet inclusion criteria (64). As a result, only their update of advice regarding fast food intake was included.

Whilst many guidelines did acknowledge the need to be mindful of the social determinants of health and their effects on the ability of families to adhere to all nutrition recommendations (APA, CAN), there were no detailed guidelines relating to cooking skills, education for food labelling, or assessment of food security within the guidelines. In fact, barriers experienced by families in line with the wider social determinants of health were generally not well addressed in the included guidelines. In order to address the known social inequalities related to the risk of childhood obesity, future guidelines should review and document outcomes for those more vulnerable children including those with disability and those from families living in disadvantage.

The importance of partaking in and increasing physical activity as part of the treatment for obesity in children and adolescents was recommended in the majority of the guidelines. However, none of the guidelines addressed methods of assessing physical activity level or the assessment of physical or social barriers that might influence participation in physical activity. Given the evidence related to the musculoskeletal and cardiorespiratory complications of obesity (106) it is important to better understand *why* a child might not reach the recommended level of daily physical activity in order to better design interventions to treat obesity and related complications. The evidence suggests that pain, obesity and a reduction in physical functioning and activity may contribute to a cycle of weight gain that affects a child’s quality of life. For example, objective assessment of cardiorespiratory fitness level can provide children, adolescents and their families with a means of understanding what moderate or vigorous activity feels like and to better prescribe appropriate fun activity to be used in treatment. Even in the absence of changes to weight status, increasing physical fitness and physical activity provides numerous health benefits to the child/adolescent including improved cardiovascular health (107), decreased risk of T2DM (108) and improved cognitive function and concentration (109), and may improve mental health and quality of life (110).

There was a dearth of information in the guidelines related to the physical functioning of children with obesity such as ability to perform activities of daily living such as dressing, toileting or showering, for which children may benefit from occupational therapist and physiotherapy support. Given the increased risk of musculoskeletal impairments, future guidelines should consider the impact of obesity on the child’s day to day functioning at home, at school and in the community. Empirical evidence suggests that compared to lean peers children with obesity have greater difficulty performing basic locomotor skills and functional tasks (111). Health outcomes related to fitness, function and quality of life are important for inclusion in core outcome sets (similar to other conditions (112)), and should be considered in further research. Use of the WHO International Classification of Functioning, Disability and Health (ICF) (113) may be a useful framework to assist development of a core outcome set for obesity assessment and treatment in children and adolescents. Detail relating to the role of nurses in the delivery of care for paediatric obesity was also missing within the included studies, yet the nursing profession has an important role in managing care of obesity and its complications across clinical settings (114).

Though many guidelines advised on using behaviour change approaches, there is little guidance around how evidence-based behaviour change models and techniques are integrated into obesity treatments. Approaches such as the components of the COM-B model and the behaviour change wheel (115) specify the various tools and techniques used to elicit change in human behaviour such as goal setting, self-monitoring and problem solving. Whilst these strategies, or their components, are often incorporated in interventions (93), few studies detail the theoretical approaches or specific tools used, and as a result, current clinical guidelines on the whole lack recommendations regarding the design and development of such complex interventions. Further, little guidance exists around circumstances where behaviour change support might need to be supplemented with more tailored psychosocial therapeutic approaches (e.g., where parental capacity is impaired or if child-parent attachment has been arrested due to adverse childhood experience).

Finally, for surgical and pharmaceutical treatments, guidelines were based on low quality evidence and a general consensus that these are not recommended in children, and only in extreme circumstances for adolescents, whereby the benefits outweigh the risks—noting that many of the risks and long-term side effects are unknown. Further evidence and recommendations are needed relating to such measures and the expertise, training, assessment, delivery, monitoring and follow-up care, as well as advice and guidance for evaluation and benchmarking of existing services and resources for delivering these.

### Implications for future research and guideline development

Future updates and development of guidelines should assess the evidence for the intensity of weight management programmes, mode of delivery, training of staff offering assessment and treatment, use of telemedicine, factors affecting engagement, managing stigma, and practical skills programmes that address the social determinants of health and account for the systemic nature of the disease. Further clarity is also needed on the use of clinical staging systems and outcomes which go beyond BMI or similar, and which can capture meaningful improvements in health, function, wellbeing, and quality of life. There is also a gap in recommendations for assessing complex obesity, or obesity as a co-morbidity/complication, versus “simple” obesity (96, 116). Guidance on screening for this may assist HCPs in identifying the most appropriate management plan. Detailed recommendations on the transition to adult services, and also related to child welfare concerns (117) in obesity assessment are needed. Multi-disciplinary stakeholder groups should be convened to oversee guideline development to ensure all perspectives are accounted for, including patient representatives, and take a social determinants of health perspective to avoid the promotion of weight stigma (118).

### Strengths and limitations

The strengths of this review of clinical guidelines were the systematic search of databases and extensive searches through reference lists and internet search engines. It is a strength that we included only those guidelines that were of high quality, and that we quantified the quality systematically, using the AGREE II tool.

It is a limitation of this review that only guidelines available in English were included and guidelines from non-English speaking regions may have been omitted. A high percentage of children/adolescents with overweight or obesity live in low- and middle-income countries (LMICs), but the guidelines included in this review, with the exception of one (WHO), were exclusively produced in high-income, English-speaking countries. For this reason, this narrative summary likely only applies to high resource settings, and guidelines from LMICs may provide recommendations that are tailored to countries where the double burden of obesity and undernutrition is present (119). This was a limitation of both our English only search, but also narrow inclusion of only documents explicitly identified as clinical practice guidelines. Further, causes and therefore appropriate management options of obesity may vary significantly in LMICs, and the included guidelines may not assess interventions that are useful for certain populations.

It is also important to note that the most recent guideline included in the review was published in 2018 and updates to guidelines are required to account for newer clinical trial data exploring interventions such as family-therapy, exercise therapy, occupational therapy, nutrition therapy, physiotherapy, pharmacotherapy and surgical procedures for obesity in children and adolescents. Further, the emergence of empirical evidence, systematic reviews, and conclusive consensus statements on treatment guidance can take years, and this highlights the importance of ‘living documents’ which are subject to frequent updates to account for emerging evidence. The guidelines synthesised in this review may no longer be underpinned by the most up to date evidence. MDTs should review the latest evidence to determine the best approach for their clinical service, whilst considering resources required and a tailored, holistic approach needed to optimise outcomes.

## Conclusion

To conclude, there is consensus that family orientated, multi-component and multi-disciplinary behaviour change interventions should be provided as the cornerstone of treatment for obesity in children and adolescents. Nine high quality guidelines are published which provide specific recommendations for the delivery of these, which vary in detail. There is insufficient evidence to provide clear guidance on the long-term effects of surgery or medication for obesity in adolescents, and these are generally not recommended for children with obesity. Future development of guidelines should consider recommendations that better address the social determinants of child and adolescent obesity. Access to evidence-based, safe, equitable care for the management of this chronic disease is vital for the prevention of complications and to ensure quality of life for all children, in line with the UN CRC.

## Data availability statement

The original contributions presented in this study are included in the article/Supplementary material, further inquiries can be directed to the corresponding author.

## Author contributions

GO’M, CW, and SB: conceptualisation, methodology, and software. GO’M, CW, LT, NA, LC, and LM: data curation, visualisation, and investigation. LT, CW, NA, SS, CO’G, AW, FW, and GO’M: writing—original draft preparation. GO’M: supervision. All authors validation and writing—reviewing and editing.

## Footnotes

1
www.agreetrust.org/my-agree
