# Supplementary material for: Guidelines for treating child and adolescent obesity: A systematic review
Source: Front Nutr. 2022 Oct 12;9:902865. doi: 10.3389/fnut.2022.902865 (PMC9597370; doi:10.3389/fnut.2022.902865)
Supplement: Supplementary file 1 [file Table_1.pdf]

Guidelines for Treating Child and Adolescent Obesity: A Systematic Review

Table S1: eligible guidelines assessed for quality

Year	Title	Publisher	Country	Method	Funding	Population
2006	Position of the American Dietetic Association: individual-, family-, school-, and community-based interventions for pediatric overweight[1]	Journal of the American Dietetic Association	USA	Systematic review	Not identified	Children (2-12 years) and adolescents (13-18 years)
2015	Pediatric Weight Management[2]	Academy of Nutrition and Dietetics	USA	Review	Not identified	Children
2018	Clinical Practice Guideline for Multicomponent Behavioral Treatment of Obesity and Overweight in Children and Adolescents[3]	American Psychological Association	USA	Clinical guidelines	American Psychological Association	Children and adolescents (2-18 years)
2005	Best Practice Guidelines in Pediatric/Adolescent Weight Loss Surgery (Apovian)[4]	Obesity Reviews	USA	Clinical guidelines	Not identified	Adolescents (12-19 years)
2019	Pediatric Metabolic and Bariatric Surgery: Evidence, barriers, and Best Practices (Armstrong)[5]	Pediatrics	USA	Systematic review	AstraZeneca	Children and adolescents (0-18 years)
2013	Clinical practice guidelines for the management of overweight and obesity in adults, adolescents and children in Australia[6]	Australian government. National Health and Medical Research Council. Department of Health	Australia	Clinical guidelines	Department of Health Australia	Children and adolescents (0-18 years) /adult
2016	Bariatric Surgery for Adolescents and Young Adults: A review of Comparative Clinical Effectiveness, Cost-effectiveness, and Evidence-Based Guidelines[7]	Canadian Agency for Drugs and Technologies in Health	Canada	Systematic review	Not identified	Children <21 years/adult

2010	The training of health professionals for the prevention and treatment of overweight and obesity[8]	Royal College of Physicians	UK	Systematic review	Not identified	Children (0-18 years)
2016	Physical therapy as treatment for childhood obesity in primary health care: Clinical recommendation from AXXON (Belgian physical therapy association). (Hansen)[9]	Physical Therapy	Belgium	Systematic review	AXXON	Children (6-12 years) and adolescents (12-18 years)
2018	Paediatric weight management evidence-based practice guidelines: Components and contexts of interventions. (Henry)[10]	Journal of the Academy of Nutrition and Dietetics	USA	Systematic review	No funding to disclose	Children (6-18 years)
2013	Position of the academy of nutrition and dietetics: Interventions for the prevention and treatment of paediatric overweight and obesity. (Hoelscher)[11]	Journal of the Academy of Nutrition and Dietetics	USA	Systematic review and position statement	Not identified	Children (2-18 years)
2008	IPEG Guidelines for Surgical Treatment of Extremely Obese Adolescents[12]	Journal of Laparoendoscopic and advanced surgical techniques	USA	Clinical guidelines	Not identified	Children
2017	Clinical Commissioning Policy: Obesity surgery for children with severe complex obesity (NHS England)[13]	NHS England	UK	Clinical guidelines	Department of Health and Social Care	Children (<18 years)
2014	Obesity: identification, assessment and management of overweight and obesity in children, young people and adults.[14]	National Institute for Health and Care Excellence (NICE)	UK	Clinical guidelines	Department of Health and Social Care	Children/adult (>2years)
2019	Current recommendations for nutritional management of overweight and obesity in children and adolescents: A structured framework (Pfeifflé)[15]	Nutrients	Switzerland	Systematic Review	HES-SO	Children (0-18 years)

2020	Childhood obesity: Evidence Based guidelines for clinical practice- Part one (Polfuss)[16]	Journal of Paediatric Healthcare	USA	Systematic review	Not identified	Children
2009	Best practice updates for paediatric/adolescent weight loss surgery (Pratt)[17]	Obesity	USA	Systematic reviews	Partly funded by the Boston Obesity Nutrition Research Center Grant P30-DK-46200 and the Center for Healthy Living, Division of Nutrition, Harvard Medical School.	Children
2018	ASMBS Paediatric metabolic and bariatric surgery guidelines (Pratt)[18]	Surgery for Obesity and Related Diseases	USA	Systematic review	Not identified	Children
2013	An overview of national clinical guidelines for the management of childhood obesity in primary care (Richardson)[19]	Preventive Medicine	Netherlands	Systematic review	Not identified	Children
2010	Management of obesity. A national clinical guideline.[20]	Scottish Intercollegiate Guidelines Network	UK	Systematic review	Not identified	Adults and Children (<18years)
2009	Clinical practice guideline for the prevention and treatment of childhood and juvenile obesity[21]	Ministry for Science and Innovation	Spain	Systematic review	Carlos III Health Institute	Children and adolescents (<18years)
2018	Recommendations for treatment of child and adolescent overweight and obesity (Spear)[22]	Paediatrics	USA	Systematic review	Not identified	Children (2-18years)
2017	Pediatric Obesity-Assessment, Treatment, and Prevention: An Endocrine Society Clinical Practice Guideline. (Styne)[23]	The Journal of Clinical Endocrinology and Metabolism	USA	Clinical guidelines	The Endocrine Society	Children

2018	Diagnosis, treatment and prevention of paediatric obesity: consensus position statement of the Italian society for paediatric endocrinology and diabetology and the Italian society of paediatrics (Valerio)[24]	Italian Journal of Paediatrics	Italy	Systematic review and consensus position statement	Not identified	Children (0–18 years)
2013	Expanding the role of primary care in the prevention and treatment of childhood obesity: A review of clinic and community-based recommendations and interventions (Vine)[25]	Journal of Obesity	USA	Systematic review	Not identified	Children
2017	Assessing and managing children at primary health-care facilities to prevent overweight and obesity in the context of double burden of malnutrition[26]	World Health Organisation	Global	Systematic review and meta-analysis.	Not identified	Adults and Children (<5 years)
2019	The management and treatment of obesity in children and youth: a briefing for European policymakers[27]	World Obesity Federation	UK	Report	European Union’s 3rd Health Programme	Children
2019	Clinical practice guideline for the diagnosis and treatment of paediatric obesity: recommendations from the committee on paediatric obesity of the Korean society of paediatric gastroenterology hepatology and nutrition (Yi)[28]	Korean Journal of Paediatrics	Korea	Systematic Review	Not identified	Children
2015	Recommendations for growth monitoring, and prevention and management of overweight and obesity in children and youth in primary care (CAN)[29]	Canadian Task Force on Preventive Health Care.	Canada	Systematic Review	Public Health Agency of Canada and the Canadian Institutes of Health Research.	Children and youth aged 2 to 17 years

Table S2: Methodological quality (AGREE II Scores, %) of the included guidelines

Guidelines	Applicability	Clarity of Presentation	Editorial Independence	Rigour of Development	Scope and Purpose	Stakeholder Involvement	Overall Assessment
American Dietetic Association[1]	10%	31%	4%	52%	42%	22%	2.5
Academy of Nutrition and Dietetics[2]	54%	58%	62%	67%	89%	69%	4.0
American Psychological Association[3]	48%	61%	79%	93%	94%	81%	6.0
Apovian[4]	38%	39%	17%	29%	50%	8%	2.0
Armstrong[5]	17%	50%	92%	50%	78%	44%	4.0
National Health and Medical Research Council, Australia[6]	92%	94%	88%	91%	92%	100%	2.5
Canadian Agency for Drugs and Technologies in Health[7]	46%	33%	33%	50%	78%	28%	3.5
Henry[10]	12%	36%	46%	30%	53%	28%	2.0
Hoelscher[11]	21%	61%	12%	43%	69%	28%	2.5
NHS surgery[13]	40%	36%	25%	25%	64%	47%	2.0
National Institute for Health and Care Excellence[14]	71%	94%	54%	94%	92%	92%	6.5
PfeifflÃ©[15]	31%	72%	67%	39%	61%	31%	2.5
Polfus[16]	10%	50%	38%	36%	53%	8%	2.0
Pratt2018[17]	21%	56%	25%	30%	50%	31%	4.5
Pratt2009[18]	12%	50%	33%	34%	50%	8%	3.0
Scottish Intercollegiate Guidelines Network[20]	69%	94%	50%	85%	92%	100%	6.5

Clinical Practice Guideline, Spain[21]	71%	78%	79%	74%	89%	72%	5.5
Spear[22]	17%	14%	25%	26%	25%	11%	1.5
Styne[23]	33%	83%	100%	72%	81%	19%	6.0
Valerio[24]	17%	78%	42%	33%	53%	17%	4.5
Vine[25]	38%	19%	21%	35%	28%	25%	3.0
World Health Organization[26]	88%	89%	96%	93%	94%	75%	6.5
Yi[28]	33%	92%	50%	55%	67%	44%	5.5
CAN[29]	58%	64%	75%	77%	64%	56%	4.0
**Mean**	**39%**	**60%**	**51%**	**55%**	**67%**	**44%**	
**Standard deviation**	**25%**	**25%**	**28%**	**24%**	**21%**	**30%**	

**Overall assessment: 1 point = lowest possible quality, 7 points = highest possible quality*

Guidelines for Treating Child and Adolescent Obesity: A Systematic Review

Supporting material: search strategy

Search ID#	Search Terms	Search Options	Last Run Via
S7	Pediatric obesity AND therapy AND (clinical guidelines or clinical practice guidelines)	Limiters - Publication Year: 2000-2020<br>Expanders - Apply equivalent subjects<br>Search modes - Boolean/Phrase	Interface - EBSCOhost Research Databases<br>Search Screen - Advanced Search<br>Database - CINAHL Plus
S6	Pediatric obesity AND therapy	Limiters - Publication Year: 2000-2020<br>Expanders - Apply equivalent subjects<br>Search modes - Boolean/Phrase	Interface - EBSCOhost Research Databases<br>Search Screen - Advanced Search<br>Database - CINAHL Plus
S5	Pediatric obesity AND therapy	Limiters - Publication Year: 2000-2020<br>Expanders - Apply equivalent subjects<br>Search modes - Boolean/Phrase	Interface - EBSCOhost Research Databases<br>Search Screen - Advanced Search<br>Database - CINAHL Plus
S4	Pediatric obesity AND therapeutics	Limiters - Publication Year: 2000-2020<br>Expanders - Apply equivalent subjects<br>Search modes - Boolean/Phrase	Interface - EBSCOhost Research Databases<br>Search Screen - Advanced Search<br>Database - CINAHL Plus
S3	Pediatric obesity AND therapy	Limiters - Publication Year: 2000-2020<br>Expanders - Apply equivalent subjects<br>Search modes - Boolean/Phrase	Interface - EBSCOhost Research Databases<br>Search Screen - Advanced Search<br>Database - CINAHL Plus
S2	Pediatric obesity AND therapy	Limiters - Publication Year: 2000-2020<br>Expanders - Apply equivalent subjects<br>Search modes - Boolean/Phrase	Interface - EBSCOhost Research Databases<br>Search Screen - Advanced Search<br>Database - CINAHL Plus
S1	Pediatric obesity AND therapy	Expanders - Apply equivalent subjects<br>Search modes - Boolean/Phrase	Interface - EBSCOhost Research Databases<br>Search Screen - Advanced Search<br>Database - CINAHL Plus

#	Query	Limiters/Expanders	Last Run Via	Results
S3	S1 AND S2	Limiters - Date of Publication: 20000101-20200531<br>Expanders - Apply equivalent subjects<br>Search modes - Boolean/Phrase	Interface - EBSCOhost Research Databases<br>Search Screen - Advanced Search<br>Database - MEDLINE;CINAHL Plus with Full Text	75
S2	clinical guideline OR clinical practice guidelines	Expanders - Apply equivalent subjects<br>Search modes - Boolean/Phrase	Interface - EBSCOhost Research Databases<br>Search Screen - Advanced Search<br>Database - MEDLINE;CINAHL Plus with Full Text	99,922
S1	pediatric obesity AND therapy	Expanders - Apply equivalent subjects<br>Search modes - Boolean/Phrase	Interface - EBSCOhost Research Databases<br>Search Screen - Advanced Search<br>Database - MEDLINE;CINAHL Plus with Full Text	4,700

Guidelines for Treating Child and Adolescent Obesity: A Systematic Review

References

1. American Dietetic Association. Position of the American Dietetic Association: individual-, family-, school-, and community-based interventions for pediatric overweight. *Journal of the American Dietetic Association.* 2006;106(6):925-945.
2. Academy of Nutrition and Dietetics. Pediatric weight management guideline. (2015) http://www.adaevidencelibrary.com/topic.cfm?cat=2721&library=EBG. [Accessed April, 2020].
3. American Psychological Association. Clinical Practice Guideline for multicomponent behavioral treatment of obesity and overweight in children and adolescents. (2018) https://www.apa.org/obesity-guideline [Accessed April, 2020].
4. Apovian CM, Baker C, Ludwig DS, Hoppin AG, Hsu G, Lenders C, Pratt JS, Forse RA, O'brien A, Tarnoff M. Best practice guidelines in pediatric/adolescent weight loss surgery. *Obesity research.* 2005;13(2):274-282.
5. Armstrong SC, Bolling CF, Michalsky MP, Reichard KW. Pediatric metabolic and bariatric surgery: Evidence, barriers, and best practices. *Pediatrics*. 2019;144(6).
6. National Health and Medical Research Council. Clinical practice guidelines for the management of overweight and obesity in adults, adolescents and children in Australia. (2013) https://www.nhmrc.gov.au/about-us/publications/clinical-practice-guidelines-management-overweight-and-obesity#block-views-block-file-attachments-content-block-1. [Accessed April, 2020].
7. Canadian Agency for Drugs and Technologies in Health. Bariatric Surgery for Adolescents and Young Adults: A Review of Comparative Clinical Effectiveness, Cost-Effectiveness, and Evidence-Based Guidelines. (2016) https://www.ncbi.nlm.nih.gov/books/NBK395803/pdf/Bookshelf_NBK395803.pdf. [Accessed April, 2020].
8. Royal College of Physicians. Report prepared for Foresight by the Royal College of Physicians. The training of health professionals for the prevention and treatment of overweight and obesity. (2010) http://www.rcplondon.ac.uk/sites/default/files/press_releases/2010/12/obesity-report-2010.pdf. [Accessed April, 2020].
9. Hansen D, Hens W, Peeters S, Wittebrood C, Van Ussel S, Verleyen D, Vissers D. Physical therapy as treatment for childhood obesity in primary health care: Clinical recommendation from AXXON (Belgian Physical Therapy Association). *Physical Therapy*. 2016;96(6):850-864.

10. Henry BW, Ziegler J, Parrott JS, Handu D. Pediatric Weight Management Evidence-Based Practice Guidelines: Components and Contexts of Interventions. *Journal of the Academy of Nutrition and Dietetics*. 2018;118(7):1301-1311.e23.
11. Hoelscher DM, Kirk S, Ritchie L, Cunningham-Sabo L. Position of the Academy of Nutrition and Dietetics: Interventions for the Prevention and Treatment of Pediatric Overweight and Obesity. *Journal of the Academy of Nutrition and Dietetics*. 2013;113(10):1375-1394.
12. International Pediatric Endosurgery Group Standards and Safety Committee. IPEG guidelines for surgical treatment of extremely obese adolescents. *Journal of Laparoendoscopic and advanced surgical techniques.* 2008;18(6):xiv-xvi.
13. National Health Service England Specialised Services Clinical Reference Group for Severe and Complex Obesity. Clinical Commissioning Policy: Obesity surgery for children with severe complex obesity. (2017) https://www.england.nhs.uk/wp-content/uploads/2017/04/16053p-obesity-surgery-children-severe-complex-obesity.pdf. [Accessed April, 2020].
14. National Institute for Health and Care Excellence. Obesity: identification, assessment and management - Clinical guideline [CG189]. (2014) https://www.nice.org.uk/guidance/cg189. [Accessed April, 2020].
15. Pfeifflé S, Pellegrino F, Kruseman M, Pijollet C, Volery M, Soguel L, Torre SBD. Current Recommendations for Nutritional Management of Overweight and Obesity in Children and Adolescents: A Structured Framework. *Nutrients*. 2019;9;11(2):362.
16. Polfuss ML, Duderstadt KG, Kilanowski JF, Thompson ME, Davis RL, Quinn M. Childhood Obesity: Evidence-Based Guidelines for Clinical Practice-Part One. *Journal Pediatric Health Care*. 2020;34(3):283-290.
17. Pratt JS, Lenders CM, Dionne EA, Hoppin AG, Hsu GL, Inge TH, Lawlor DF, Marino MF, Meyers AF, Rosenblum JL, Sanchez VM. Best practice updates for pediatric/adolescent weight loss surgery. *Obesity (Silver Spring).* 2009;17(5):901-10.
18. Pratt JSA, Browne A, Browne NT, Bruzoni M, Cohen M, Desai A, Inge T, Linden BC, Mattar SG, Michalsky M, Podkameni D, Reichard KW, Stanford FC, Zeller MH, Zitsman J. ASMBS pediatric metabolic and bariatric surgery guidelines, 2018. *Surgery for obesity and related diseases: official journal of the American Society for Bariatric Surgery*. 2018;14(7):882-901.
19. Richardson LA, Paulis WD, van Middelkoop M, Koes BW. An overview of national clinical guidelines for the management of childhood obesity in primary care. *Preventive medicine.* 2013;57(5), 448-55.

20. Scottish Intercollegiate Guidelines Network SIGN 115. Management of obesity: A National Clinical Guideline. (2010) https://www.sign.ac.uk/assets/sign115.pdf. [Accessed April, 2020].
21. Working Group of the Guideline for the Prevention and Treatment of Childhood and Juvenile Obesity; Iberoamerican Cochrane Centre, coordinator. Clinical Practice Guideline for the Prevention and Treatment of Childhood and Juvenile Obesity. (2009) https://portal.guiasalud.es/wp-content/uploads/2019/01/GPC_452_obes_infantojuv_AATRM_compl_en.pdf. [Accessed April, 2020].
22. Spear BA, Barlow SE, Ervin C, Ludwig DS, Saelens BE, Schetzina KE, Taveras EM. Recommendations for treatment of child and adolescent overweight and obesity. *Pediatrics*. 2007;120(4):254-88.
23. Styne DM, Arslanian SA, Connor EL, Farooqi IS, Murad MH, Silverstein JH, Yanovski JA. Pediatric Obesity-Assessment, Treatment, and Prevention: An Endocrine Society Clinical Practice Guideline. *The Journal of clinical endocrinology and metabolism*. 2017;102(3):709-757.
24. Valerio G, Maffeis C, Saggese G, Ambruzzi MA, Balsamo A, Bellone S, Bergamini M, Bernasconi S, Bona G, Calcaterra V, Canali T, Caroli M, Chiarelli F, Corciulo N, Crinò A, Di Bonito P, Di Pietrantonio V, Di Pietro M, Di Sessa A, Diamanti A, Doria M, Fintini D, Franceschi R, Franzese A, Giussani M, Grugni G, Iafusco D, Iughetti L, Lamborghini A, Licenziati MR, Limauro R, Maltoni G, Manco M, Reggiani LM, Marcovecchio L, Marsciani A, Del Giudice EM, Morandi A, Morino G, Moro B, Nobili V, Perrone L, Picca M, Pietrobelli A, Privitera F, Purromuto S, Ragusa L, Ricotti R, Santamaria F, Sartori C, Stilli S, Street ME, Tanas R, Trifiró G, Umano GR, Vania A, Verduci E, Zito E. Diagnosis, treatment and prevention of pediatric obesity: consensus position statement of the Italian Society for Pediatric Endocrinology and Diabetology and the Italian Society of Pediatrics. *Italian journal of pediatrics*. 2018;31;44(1):88.
25. Vine M, Hargreaves MB, Briefel RR, Orfield C. Expanding the role of primary care in the prevention and treatment of childhood obesity: a review of clinic- and community-based recommendations and interventions. *Journal of obesity*. 2013;172035.
26. World Health Organization. Guideline: assessing and managing children at primary health-care facilities to prevent overweight and obesity in the context of the double burden of malnutrition. Updates for the Integrated Management of Childhood Illness (IMCI). (2017) https://apps.who.int/nutrition/publications/guidelines/children-primaryhealthcare-obesity-dbm/en/index.html. [Accessed April, 2020].
27. World Obesity Federation. The management and treatment of obesity in children and youth: a briefing for European policymakers. (2019) https://s3-eu-west-1.amazonaws.com/wof-files/CHOTreatment_Briefing.pdf. [Accessed April, 2020].

28. Yi DY, Kim SC, Lee JH, Lee EH, Kim JY, Kim YJ, Kang KS, Hong J, Shim JO, Lee Y, Kang B, Lee YJ, Kim MJ, Moon JS, Koh H, You J, Kwak YS, Lim H, Yang HR. Clinical practice guideline for the diagnosis and treatment of pediatric obesity: recommendations from the Committee on Pediatric Obesity of the Korean Society of Pediatric Gastroenterology Hepatology and Nutrition. *Korean journal of paediatrics*. 2019; 62(1):3-21.
29. Canadian Task Force on Preventive Health Care. Recommendations for growth monitoring, and prevention and management of overweight and obesity in children and youth in primary care. *Canadian Medical Association Journal*. 2015;187(6):411-421.
